# Emotional Blunting During Antidepressant Treatment: Implications for Clinical Decision‐Making and Patient‐Centred Care

**DOI:** 10.1111/jep.70604

**Published:** 2026-10-06

**Authors:** Carlos De las Cuevas

**Affiliations:** ^1^ Department of Internal Medicine, Dermatology and Psychiatry University of La Laguna Tenerife Spain

**Keywords:** antidepressants, emotional blunting, long‐term treatment, patient‐reported outcomes, shared decision‐making

## Abstract

**Rationale:**

Emotional blunting is frequently reported during antidepressant treatment but remains conceptually underdefined. Reduced emotional reactivity and affective intensity may represent therapeutic benefit, an adverse effect, residual depressive symptomatology, or a context‐dependent combination of these possibilities.

**Aims and Objectives:**

To clarify the phenomenology and clinical significance of antidepressant‐associated emotional blunting, distinguish it from residual depression, anhedonia, and apathy, and consider its implications for long‐term prescribing, shared decision‐making, and patient‐centred care.

**Method:**

Evidence from phenomenological and qualitative accounts, survey‐based and clinical studies, neurobiological research, patient‐reported outcome literature, and relevant ethical and clinical guidance was narratively integrated to examine the prevalence, mechanisms, subjective meanings, and management of emotional blunting.

**Results:**

Emotional blunting is reported by a substantial proportion of antidepressant‐treated patients, although estimates vary because of differences in definitions, instruments, populations, and study designs. Evidence suggests that serotonergic antidepressants may dampen the processing of both rewarding and aversive stimuli, but medication effects cannot always be clearly separated from residual illness. Patients' appraisals are heterogeneous: emotional attenuation may be experienced as protective or acceptable during acute treatment yet burdensome, alienating, or inconsistent with personal recovery during maintenance treatment. Misclassification may lead to inappropriate dose escalation, unnecessary continuation, or premature discontinuation. Clinical responses should therefore be individualised and may include maintaining treatment, dose reduction, switching, psychological or psychosocial interventions, or planned discontinuation.

**Conclusion:**

Emotional blunting is best understood as a therapeutically ambivalent, patient‐reported outcome whose significance varies with illness phase, treatment goals, perceived benefits, and individual values. Systematic enquiry and shared decision‐making can support more clinically appropriate and person‐centred antidepressant care.

## 1 Introduction

Antidepressant prescribing has become a defining feature of contemporary psychiatric and primary‐care practice. Across OECD countries, population‐level data indicate a sustained rise in antidepressant consumption over the past decade, with an increase of approximately 50% between 2011 and 2021 and marked inter‐country variability [1, 2]. This growth reflects multiple converging factors, including broader diagnostic recognition, expanded therapeutic indications, evolving clinical guidelines, and, in particular, the increasing prevalence of long‐term and maintenance prescribing. In routine clinical settings, a substantial proportion of patients continue antidepressant treatment well beyond the acute phase of illness, either as prophylaxis for recurrent depression or for non‐depressive conditions such as anxiety disorders [3]. Against this background, this article examines emotional blunting as a clinically and ethically relevant outcome of long‐term antidepressant treatment. Understanding this phenomenon has direct implications for everyday clinical decision‐making. However, its interpretation in routine clinical practice remains uncertain and often varies between clinicians.

In this context, increasing attention has been directed towards experiential outcomes of antidepressant treatment that are meaningful for patients but often remain insufficiently captured by conventional symptom‐based outcome measures.

Long‐term antidepressant exposure has also attracted increasing attention because of growing evidence regarding withdrawal phenomena following discontinuation. Systematic reviews and large patient surveys suggest that withdrawal symptoms may occur in a substantial proportion of antidepressant users and can vary widely in severity and duration [4]. These findings have prompted renewed debate about the long‐term balance of benefits and harms of antidepressant treatment and about the adequacy of current clinical guidelines in addressing withdrawal risks [5]. Recognising these broader concerns is important when evaluating experiential outcomes such as emotional blunting, which may influence patients' attitudes toward continuing or discontinuing long‐term treatment.

This shift towards prolonged exposure has sharpened attention to outcomes that extend beyond symptom reduction. Although antidepressant efficacy has traditionally been evaluated using symptom‐based rating scales, patients frequently report treatment effects that are not adequately captured by these measures. These include changes in emotional experience, interpersonal engagement, motivation, and perceived sense of self [6]. Among these experiential outcomes, emotional blunting—commonly described as a subjective attenuation in the intensity and range of both positive and negative emotions—has emerged as a particularly salient and clinically relevant phenomenon.

Emotional blunting is not uncommon. Qualitative interviews and survey‐based studies consistently indicate that a substantial proportion of patients treated with antidepressants report feeling emotionally ‘numb’, ‘flat’, or detached [7, 8, 9, 10].

Importantly, such experiences often persist despite symptomatic remission and may influence quality of life, treatment satisfaction, and treatment decisions [9, 10, 11, 12]. Nevertheless, emotional blunting occupies an uncertain conceptual position within psychiatry. It has been variously framed as an adverse drug effect, a manifestation of residual depressive pathology, or a benign—or for some patients temporarily acceptable or even valued—by‐product of pharmacological emotional regulation.

Patients' interpretations of emotional blunting vary considerably. While many describe the experience as troubling or alienating, others report perceiving it as a temporary trade‐off that accompanies relief from overwhelming negative affect. Recent clinical discussions have emphasised that emotional blunting remains an under‐recognised but clinically significant experience associated with antidepressant treatment and warrants greater attention in both research and clinical practice [13].

This conceptual ambiguity carries tangible clinical consequences. When emotional blunting is interpreted as evidence of incomplete recovery, clinicians may respond by escalating doses or prolonging pharmacological treatment. Conversely, when it is viewed solely as an adverse effect, dose reduction, switching, or discontinuation may be prioritised, potentially increasing relapse risk. In practice, emotional blunting frequently occupies an intermediate position: it may reflect a complex interplay between medication effects and illness‐related processes, and its subjective significance varies widely across individuals, illness phases, and treatment contexts.

In this article, we propose that emotional blunting associated with antidepressant treatment is best understood as a therapeutically ambivalent outcome whose clinical significance cannot be determined independently of patient values, illness trajectory, and treatment goals. By synthesising empirical findings, neurobiological evidence, and clinical reasoning, we aim to clarify the relevance of emotional blunting for long‐term antidepressant prescribing, to delineate its distinction from related constructs such as anhedonia and apathy, and to outline a pragmatic, patient‐centred approach to assessment and shared decision‐making in routine clinical practice.

In a context of widespread long‐term and maintenance antidepressant prescribing, clarifying the clinical meaning of emotional blunting is increasingly relevant for reflective, patient‐centred decision‐making.

Such situations exemplify the need for interpretative clinical reasoning in contexts where patient experience does not align straightforwardly with conventional outcome measures.

From a clinical perspective, emotional blunting represents not only a descriptive phenomenon but also a challenge for decision‐making. Clinicians are required to determine whether it reflects therapeutic benefit, adverse effect, or an acceptable trade‐off, often in the absence of clear guidance. This interpretative uncertainty highlights the role of clinical reasoning in integrating patient‐reported experiences into treatment decisions and makes emotional blunting a useful model for examining how such experiences are understood in everyday practice.

## 2 Defining Emotional Blunting: Phenomenology and Differentiation

Emotional blunting refers to a subjective reduction in emotional responsiveness, often described as a diminished capacity to experience both positive and negative emotions. Patients frequently report feeling emotionally ‘flattened’, detached, or less able to react to events that would previously have elicited strong emotional responses [7]. These experiences may overlap with related constructs such as apathy or diminished motivation, which can occur during antidepressant treatment and may share partially overlapping neurobiological mechanisms [14].

Residual depressive symptoms typically include persistent low mood, cognitive distortions, fatigue, or impaired concentration and are usually experienced as distressing. Anhedonia, by contrast, refers more specifically to a reduced capacity to experience pleasure in response to rewarding stimuli and constitutes a core symptom of depressive disorders [15]. Apathy is characterised primarily by diminished motivation or initiative rather than a qualitative alteration in emotional intensity and may occur with limited subjective distress [14, 16]. Although these phenomena may coexist with emotional blunting, they are not phenomenologically equivalent and should not be treated as interchangeable. For clarity, key phenomenological and clinical distinctions between emotional blunting and related constructs are summarised in Table 1.

**TABLE 1:** Phenomenological and clinical differentiation of emotional blunting and related constructs.

Feature	Emotional blunting	Residual depression	Anhedonia	Apathy
Core experience	Attenuation of emotional intensity (positive and negative affect)	Persistent low mood and/or cognitive depressive symptoms	Reduced capacity to experience pleasure	Reduced motivation or initiative
Subjective distress	Variable; may be neutral or contextually valued	Usually distressing	Usually distressing	Often limited subjective distress
Temporal relation to antidepressant treatment	Frequently emerges after initiation or dose increase	Persists despite treatment	Often present prior to treatment	May be illness‐related or medication‐associated
Positive emotions	Diminished	Diminished	Markedly reduced or absent	Preserved but weakly motivating
Negative emotions	Also attenuated	Persistently present	Variable	Variable
Impact on sense of self/identity	Frequently reported	Less prominent	Moderate	Usually minimal
Change with dose adjustment	May improve with dose reduction	May worsen	Unpredictable	May improve
Patient appraisal	Variable; context‐ and value‐dependent	Predominantly negative	Predominantly negative	Often neutral

*Note:* Misclassification of emotional blunting as residual depression, anhedonia, or apathy may lead to inappropriate dose escalation, unnecessary continuation of treatment, or premature discontinuation.

Emotional blunting partially overlaps with these domains but is not reducible to any single one of them. Notably, patients may report emotional blunting in the absence of low mood and frequently describe it as qualitatively different from the emotional experience of depression itself [7, 9]. This distinction has important clinical implications. When emotional blunting is misattributed to residual depressive pathology, clinicians may escalate doses or prolong pharmacological treatment unnecessarily. Conversely, recognising emotional blunting as a medication‐associated experiential change opens alternative management strategies, including dose adjustment, switching, or structured discussion of patient preferences.

At the same time, emotional blunting should not be conceptualised as a unitary or isolated phenomenon. Rather, it lies within a broader phenomenological spectrum of emotional attenuation. In both clinical practice and research, terms such as affective flattening, emotional numbness, anhedonia, apathy, and loss of emotional responsiveness are often used to describe overlapping aspects of this experience. These constructs emerge from different theoretical traditions—classical psychopathology (affective flattening), motivational neuroscience (anhedonia and apathy), and patient‐reported experiential language (emotional numbness)—yet they converge on a shared experiential core characterised by reduced emotional intensity and reactivity.

This conceptual and terminological overlap has implications beyond phenomenology. Several of these terms are variably represented as Preferred Terms within MedDRA [17], leading to heterogeneous coding of similar patient experiences in pharmacovigilance databases such as VigiBase, maintained by the WHO Uppsala Monitoring Centre. As a result, comparable clinical phenomena may be classified under different labels depending on reporter language, coding conventions, or regulatory context. Recognising this heterogeneity is essential to improve interpretability of both clinical and pharmacovigilance data and to avoid the erroneous attribution of emotional blunting exclusively either to residual illness or to discrete adverse drug effects.

The development of the Oxford Questionnaire on the Emotional Side‐effects of Antidepressants (OQuESA) represented an important step towards operationalising emotional blunting as a measurable patient‐reported outcome [8]. The instrument captures multiple dimensions of emotional change, including emotional detachment, reduced positive affect, and general emotional dampening, and has demonstrated acceptable psychometric properties. Its use underscores the legitimacy of emotional blunting as a distinct experiential outcome while explicitly acknowledging its partial overlap with adjacent emotional and motivational constructs.

## 3 Prevalence and Clinical Correlates

Available evidence indicates that emotional blunting is a common experience among patients treated with antidepressants, particularly selective serotonin reuptake inhibitors and serotonin–noradrenaline reuptake inhibitors. Most data derive from cross‐sectional surveys and qualitative studies rather than from prospective trials specifically designed to capture this outcome. Within these constraints, reported prevalence estimates typically range from approximately 40% to over 60%, with substantial variability, according to study design, assessment instruments, and patient populations [9, 10, 18].

Large international surveys provide more precise estimates. However, survey‐based studies may overestimate prevalence due to self‐selection and recall bias. In a study involving 1829 antidepressant users, Read and colleagues reported emotional blunting in approximately 60% of participants, while a later international survey including 953 respondents found a prevalence of 71%, with emotional blunting emerging as one of the most frequently reported adverse experiences associated with antidepressant use [10, 18]. These studies also suggested that emotional blunting may be more commonly reported among younger individuals and may occur independently of treatment duration or gender. More recent literature has reinforced the clinical relevance of this phenomenon and has highlighted its heterogeneous presentation across depressive populations and treatment contexts [19, 20].

In one of the largest survey‐based studies to date, Goodwin and colleagues found that nearly half of patients receiving antidepressant treatment reported emotional blunting. Subjective appraisal was heterogeneous: although many participants experienced emotional blunting as distressing, a substantial minority described it as neutral or even beneficial [9]. This variability underscores that prevalence figures alone are insufficient to capture the clinical significance of emotional blunting and that patient interpretation plays a central role in determining its impact.

Notably, emotional blunting has been reported not only during acute treatment phases but also during long‐term and maintenance therapy, including among patients who otherwise meet conventional criteria for remission [7, 9]. This observation raises important questions about the adequacy of outcome frameworks that equate symptomatic remission with optimal recovery. Emotional blunting highlights a potential disconnect between clinician‐rated symptom resolution and patient‐experienced well‐being, particularly in the context of prolonged antidepressant use.

Associations between emotional blunting and treatment adherence further illustrate its clinical relevance. For some patients, emotional blunting contributes to dissatisfaction with medication and motivates dose reduction or discontinuation, at times without medical supervision [9, 10]. For others—especially individuals with histories of severe anxiety, affective instability, or emotional dysregulation—emotional blunting may be experienced as protective and may reinforce adherence to long‐term treatment [9].

Taken together, these findings caution against uniform assumptions regarding the desirability or undesirability of emotional blunting. Rather than constituting a homogeneous adverse outcome, emotional blunting appears to function as a context‐dependent clinical phenomenon whose significance emerges through its interaction with individual vulnerability, illness trajectory, and patient priorities.

## 4 Neurobiological Plausibility

Although emotional blunting is inherently a subjective and phenomenological experience, converging lines of evidence support plausible neurobiological mechanisms by which antidepressant treatment may alter emotional processing. Experimental studies in healthy volunteers have shown that short‐term administration of selective serotonin reuptake inhibitors can attenuate neural responses to both rewarding and aversive stimuli, consistent with a reduction in emotional salience rather than a selective effect limited to negative affect [21, 22].

In patients with depression, antidepressant treatment has been shown to modify the neural processing of emotional information early in the course of therapy. Functional neuroimaging studies have consistently shown a normalisation of heightened limbic reactivity—particularly within the amygdala—to negative emotional stimuli, often preceding overt changes in mood or subjective symptom improvement [23, 24]. While these effects are commonly interpreted as core mechanisms of antidepressant efficacy, they also provide a plausible pathway through which emotional responsiveness may be modulated or attenuated, potentially contributing to experiences of emotional blunting.

These neurobiological findings should not be interpreted deterministically. Demonstrating that antidepressants can modulate emotional salience at the neural level does not imply that emotional blunting is inevitable, uniform, or exclusively medication‐induced. Individual vulnerability, illness characteristics, baseline emotional reactivity, dosage, and duration of treatment are all likely to shape whether and how such neurobiological changes are subjectively experienced.

Nevertheless, the presence of biologically plausible mechanisms lends credibility to patient reports of emotional blunting and argues against their dismissal as merely psychological or nonspecific, or attributable solely to residual depressive pathology. Emotional blunting may therefore represent a downstream experiential consequence of neurobiological mechanisms that are therapeutically beneficial for some patients, while being perceived by others as constraining or alienating depending on illness phase and personal meaning.

An alternative interpretative framework has been proposed within the so‐called 'drug‐centred' model of psychotropic drug action. According to this perspective, antidepressants are understood primarily as agents that induce altered mental and physiological states rather than as treatments that specifically correct underlying disease processes. Within this framework, emotional blunting may be interpreted as part of a broader drug‐induced modification of emotional and motivational functioning, potentially linked to other commonly reported effects such as apathy, lethargy, or sexual dysfunction [25, 26, 27]. Although this model remains the subject of considerable debate, acknowledging it highlights the broader conceptual uncertainty surrounding the mechanisms through which antidepressants influence emotional experience and underscores the need for further empirical investigation. Empirical evidence directly comparing disease‐centred and drug‐centred interpretations of antidepressant effects remains limited.

## 5 Therapeutic Ambivalence and Patient Meaning

The most distinctive feature of emotional blunting in clinical practice is its therapeutically ambivalent meaning. Qualitative studies consistently suggest that patients do not experience emotional blunting in a uniform or unidirectional manner [7]. For some individuals, emotional blunting represents an unwelcome loss of vitality, pleasure, and emotional authenticity, often articulated as feeling ‘less like oneself’, a recurrent theme in sociological and anthropological accounts of illness experience [28, 29]. For others—particularly during periods of acute distress—emotional blunting may be welcomed as a relief from overwhelming affect, intrusive rumination, or anxiety [7].

Importantly, the same individual may experience emotional blunting differently across time and illness phases. During acute episodes, a reduction in emotional intensity may facilitate stabilisation and functional recovery. During prolonged remission or maintenance treatment, however, the same experiential change may come to be perceived as constraining, alienating, or incompatible with personal identity. This temporal dimension is often overlooked in static assessments of treatment outcomes but is central to understanding patient attitudes towards long‐term antidepressant use and personal recovery [30].

The dynamic and context‐dependent clinical meaning of emotional blunting across treatment phases is summarised in Figure 1.

**FIGURE 1:**
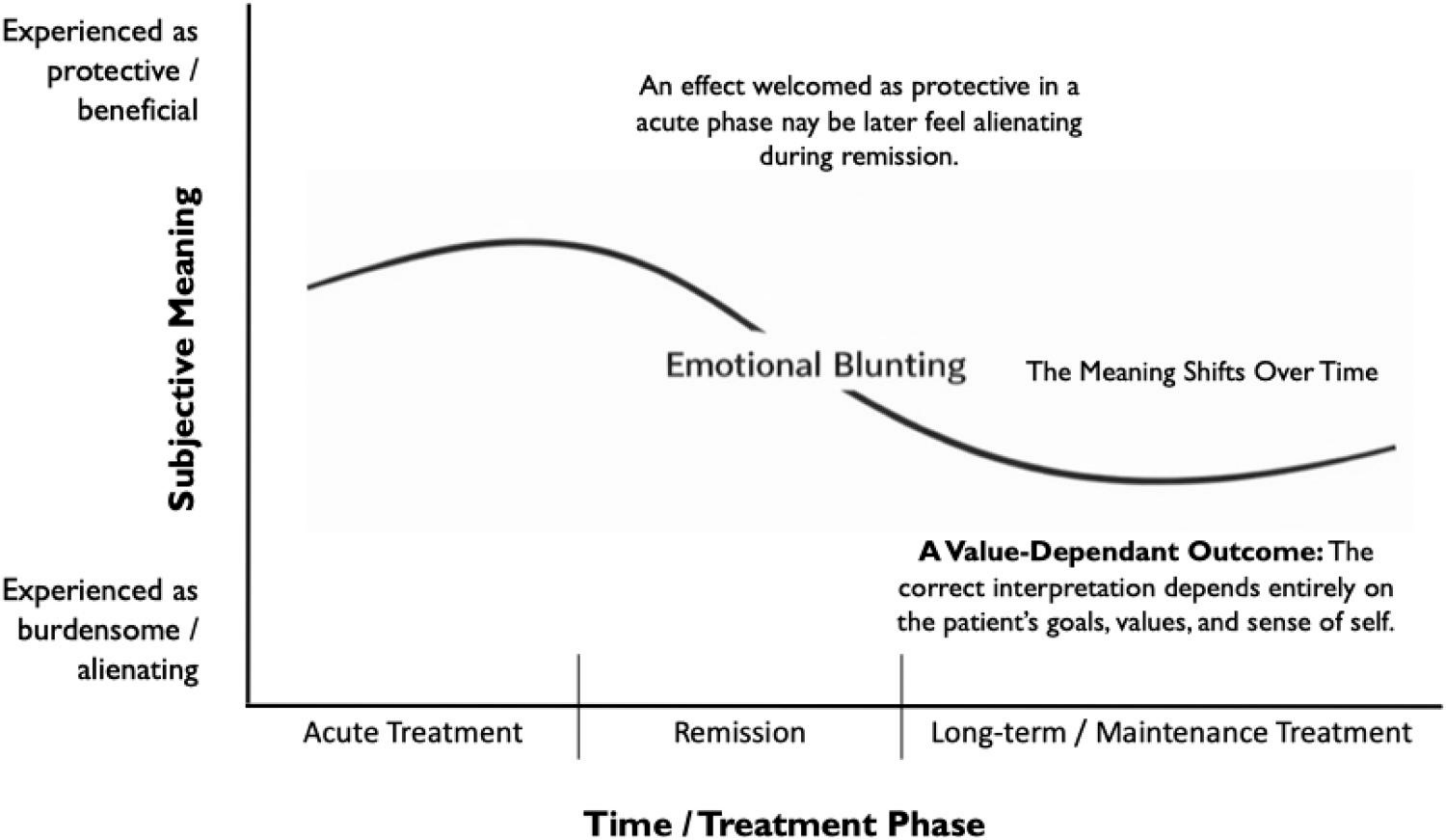
Emotional blunting as a therapeutically ambivalent outcome across treatment phases. The figure illustrates how the subjective meaning of emotional blunting may shift over time, being experienced as protective or beneficial during acute treatment and as burdensome or alienating during prolonged remission or maintenance, depending on clinical context and patient values.

From a clinical standpoint, emotional blunting therefore resists categorical classification as either an adverse effect or a therapeutic benefit. Its significance emerges through dialogue with the patient and depends on how it aligns—or conflicts—with individual goals, values, and sense of self. What may be experienced as protective at one stage of illness may later be perceived as limiting or unacceptable, particularly when treatment continues beyond the resolution of acute symptoms.

Recognising this therapeutic ambivalence challenges conventional outcome hierarchies that privilege symptom reduction as the primary marker of success. Emotional blunting highlights the limits of symptom‐centric models of recovery and remission and underscores the importance of attending to subjective well‐being, personal meaning, and identity‐related outcomes [30]. Incorporating these dimensions into clinical reasoning invites a more nuanced and ethically grounded approach to antidepressant prescribing, particularly in the context of long‐term and preventive treatment.

## 6 Clinical Implications for Decision‐Making

The therapeutically ambivalent nature of emotional blunting has important clinical and ethical implications, particularly in the context of long‐term antidepressant treatment. In routine practice, clinicians are often required to interpret such experiences in order to guide treatment decisions. Importantly, emotional blunting should not automatically be interpreted as either therapeutic failure or medication toxicity. Rather, its clinical significance depends on how the experience is perceived by the patient and how it relates to the goals and stage of treatment.

First, emotional blunting may be explicitly acknowledged and discussed with patients as a potential experiential outcome of antidepressant therapy, especially when treatment is expected to extend beyond the acute phase of illness. Limited recognition or discussion of this possibility may complicate processes of informed consent and may affect how patients interpret changes in their emotional experience [31]. This process is inherently interpretative and depends on how clinicians integrate patient‐reported experiences into their clinical reasoning.

Second, emotional blunting warrants active and systematic attention during follow‐up, rather than being addressed only when patients spontaneously report distress. Because emotional attenuation may be normalised, minimised, or difficult to articulate, neutral and open‐ended enquiry is often required to elicit these experiences. Where feasible, brief patient‐reported measures—such as selected items from the Oxford Questionnaire on the Emotional Side‐effects of Antidepressants—may support more structured exploration without medicalising the experience or presuming distress [8].

Emotional blunting may also coexist with other medication‐associated experiential changes, including sexual dysfunction, apathy, or reduced motivational drive [16, 32, 33, 34]. Increasing attention has been drawn to reports of persistent sexual dysfunction following discontinuation of serotonergic antidepressants, although the prevalence and causal mechanisms remain uncertain. While the relationship between these phenomena and emotional blunting is not yet fully understood, their potential co‐occurrence reinforces the importance of discussing a broad range of possible experiential outcomes when initiating or maintaining antidepressant therapy.

Third, decisions regarding the management of emotional blunting should be guided by shared decision‐making principles [35, 36]. Potential strategies include maintaining the current regimen when emotional blunting is acceptable or valued, dose reduction, switching to antidepressants with different emotional profiles, or integrating psychological and psychosocial interventions. In some circumstances, particularly when treatment is primarily preventive and emotional blunting is experienced as burdensome, a planned and monitored discontinuation may be an ethically defensible option, consistent with contemporary clinical guidelines [37].

There is no universally correct response to emotional blunting. The ethical imperative lies not in enforcing a predetermined notion of optimal outcome, but in aligning treatment with the patient's informed preferences, evolving goals, and sense of self over time. Attending to emotional blunting in this way shifts clinical focus from symptom control alone towards a more person‐centred and ethically grounded conception of long‐term antidepressant care [31, 35]. In this sense, emotional blunting may function as a clinically informative signal that requires interpretation rather than a predefined response.

## 7 Conclusion and Future Directions

Despite growing recognition of emotional blunting in clinical practice, important gaps remain in the evidence base. Longitudinal studies are needed to characterise the trajectory of emotional blunting over time, particularly among patients in symptomatic remission who continue antidepressant treatment for maintenance or preventive treatment. Such designs would help clarify whether emotional blunting represents a stable trait, a fluctuating state, or a time‐limited phenomenon that evolves along the illness course and treatment duration, addressing longstanding limitations of symptom‐based outcome frameworks [6, 38].

Greater integration of patient‐reported outcomes into antidepressant trials is also warranted. While symptom‐based scales remain essential, they provide an incomplete account of treatment impact, particularly in long‐term use [6]. Incorporating validated measures of emotional experience would allow a more balanced evaluation of benefits and harms and better reflect outcomes that patients themselves prioritise [39]. Comparative studies across antidepressant classes, doses, and treatment strategies may further help to identify differential emotional profiles, informing more individualised prescribing decisions. Recent work also suggests that emotional blunting may have measurable consequences for social functioning and depressive symptom burden, underlining the importance of integrating functional and patient‐valued outcomes into future research [40].

Qualitative research has a complementary role in elucidating how patients interpret emotional changes over time and how these interpretations influence adherence, satisfaction, and treatment decisions. Understanding the meanings patients ascribe to emotional blunting—rather than merely its presence or absence—is particularly important when evaluating complex interventions and longitudinal treatment strategies [41]. Such approaches may also enrich shared decision‐making processes during maintenance treatment.

Ultimately, advancing research in this area requires moving beyond symptom‐centric models of outcome towards a broader conception of recovery that incorporates subjective well‐being, emotional vitality, and personal meaning [37]. Recognising emotional blunting not as a failure of treatment but as a clinically meaningful signal may help reframe long‐term antidepressant care in more ethically and person‐centred terms. This perspective may also offer a useful framework for addressing other experiential outcomes in psychopharmacology that challenge conventional evaluative models.

## Conflicts of Interest

The author declares no conflicts of interest.

## Data Availability

Data sharing not applicable to this article as no datasets were generated or analysed during the current study.
